# Measurement Method for the Simultaneous DeterMination of Thermal Resistance and Temperature Gradients in the DeterMination of Thermal Properties of Textile Material Layers

**DOI:** 10.3390/ma14226853

**Published:** 2021-11-13

**Authors:** Snježana Firšt Rogale, Dubravko Rogale, Željko Knezić, Nikolina Jukl

**Affiliations:** 1 Department of Clothing Technology, Faculty of Textile Technology, University of Zagreb, 10000 Zagreb, Croatia; sfrogale@ttf.unizg.hr; 2 Department of Textile Design and Management, Faculty of Textile Technology, University of Zagreb, 10000 Zagreb, Croatia; zeljko.knezic@ttf.unizg.hr; 3 Konfeks, 10000 Zagreb, Croatia; nikolina.jukl@gmail.com

**Keywords:** thermal resistance, temperature gradient, textile material layers, composite clothing

## Abstract

The thermal properties of most clothing products are still not designed according to engineering science due to the lack of simple and acceptable measuring equipment and methods; the type of thermal insulation material, the number of layers of clothing and their thickness are thus chosen empirically. The novelty of this study was the development of a new measuring device and method for simultaneous measurements in the determination of the thermal resistance in one or more textile material layers, such as in multilayer composite clothing. Temperature gradients of textile material layers are presented, as well as the theoretical principles of operation and practical results. Four materials for the production of protective jackets were selected, from which different combinations of composite clothing were constructed and the thermal parameters were measured with a new method and a new device, both individually for the built-in materials and for the composites. Subsequently, five test jackets with the same arrangement of textile material layers as the previously tested composites were produced, and measurements of important thermal parameters were recorded with a thermal mannequin. The determined temperature gradients and measurement results are presented, and based on these it was determined that the total thermal resistance was not equal to the algebraic sum of the resistances of the individual textile material layers in the horizontal position; it was, however, higher, increasing from 30% to 94% due to small air layers caused by crimping and protruding fibres of yarn in the textile fabrics. The same textile material layers built into clothing in the vertical position allowed the formation of significantly wider air layers that increased the thermal resistance by between 2.5 and 9 times.

## 1. Introduction

The dominant role of clothing has always been thermal protection, and throughout history clothing has been made of materials that have different levels of thermal insulation. Since ancient times, people have dressed in layers, and the lower the ambient temperature is, the more layers are used. Research has shown that the thermal properties of clothing are greatly influenced by the environmental factor, clothing factor and human factor and, considering these factors, approximate values for the thermal protection of individual clothing types or clothing systems can be determined. When buying clothes that are primarily intended for protection against the cold (winter coats, wind-jackets, coats, sweaters, pullovers, protective clothing, etc.), there is still no exact method for evaluating an item of clothing in terms of a precisely measured degree of thermal protection. The buyer therefore has to rely on buying the garment according to their visual impression and their experience assessment of the construction of the clothing, the material thickness and the raw material composition, without knowing the actual thermal protection characteristics. Currently, some clothing items and accessories (e.g., sleeping bags, sky jackets), along with other clothing labels, include wind-chill calculations but do not refer to the values for the thermal insulation of the clothing. Similarly, if the buyer hesitates between two garments they want to buy, they cannot come close to determining which has better thermal insulation properties. Thus, it is common today to see a clothing-size designation or some other body measurement that further characterises the clothing on the hang tags of clothing, but a specific thermal parameter that would provide some accurate data about the thermal properties of the clothing, e.g., the value of the thermal resistance of the clothing, the thermal conductivity of the clothing, the thermal insulation, etc., is never shown. Therefore, when buying clothes and choosing between several different items of clothing, it is not possible to choose the clothing with the best thermal insulation properties. Even during the engineering design of new clothing, it is not possible to complete the exact technical design of clothing if the thermal parameters of the built-in layers that make up the composites (one or more layers of different joined and/or embedded textiles and/or other materials) are not known, and it is also difficult to determine the success of embedding these composites, the selection of materials and their thicknesses, joints, thread density and finishing, as well as the clothing cut and the success of the general construction of clothes and cuts with regard to the final thermal properties of newly designed and manufactured items of clothing.

The current difficulties in determining the thermal properties of composites and clothing have been partially solved by introducing so-called thermal mannequins, but other necessary measuring devices are still in the development phase and are therefore not yet widely used.

The assessment of the thermal protection of an individual clothing item or clothing system can also be undertaken through subjective assessment by the wearer or through exact measurements using measuring systems intended for testing the thermal properties of textiles, textile material layers and multilayer composite clothing. However, when buying clothes, the customer still does not know the exact or even approximate value of the thermal protection of the clothes. In addition to the other information on labels sewn-in or attached to clothing, such as the size number, raw material composition and the type of care required for the clothing, the value for the thermal protection of the clothing should also be indicated.

Previous studies on the thermal properties of clothing include research on heat transfer in textile materials and the impact of the air layers in the textile materials themselves and between the thermal insulation layers. 

Thus, Mell conducted an extensive study using a model of heat conduction through textile materials (a heat-transfer model) and specifically addressed the importance of air layers as efficient thermal insulation in firefighting clothing [1]. 

Likewise, Miedzińska investigated the effects of airbags in textile structures intended for the production of firefighters’ protective clothing on the temperature distribution in the layers that occurs when the surface layer is heated to 300 °C. Textile structures consist of an outer layer, a membrane, a thermal insulation layer and lining. The author provided a numerical model of the temperature gradient distribution in the sample at different mesh sizes, the temperature gradient distribution in the sample for models with different air layer thicknesses, and a comparison of temperature gradient distributions in the sample for models with different shapes for the airlock. The results showed that increasing the thickness of the air gap increased the temperature gradients in such a way that the lowest temperature gradient was near the firefighter’s body [2]. 

Similar research has been conducted by Schmid et al. [3]. They studied the influence of critical heat transfer through multilayered firefighting clothing during a flame engulfment test. However, they did not investigate the temperature gradients between the layers. 

Gupta also investigated the influence of air-gap thickness on the thermal resistance of two-layer fabrics, but the study did not include the temperature gradient [4]. 

Sun et al. [5] investigated the heat transfer across textile layers using mathematical models and the finite element method. They also concluded that the size of air gaps has a significant impact on heat transfer. The equilibrium heat flux decreased by 40% when the air gap increased from 2 to 10 mm. Furthermore, they found that the number of layers in a textile multilayer assembly has a greater impact when the ambient temperature is lower. They showed the heat flux distribution against time but not the temperature gradients.

Onofrei et al. [6], in their research, also presented numerical predictions of the temperature distribution in fabric as a function of time.

Venkataraman stated that the thermal conductivity is approximately constant for a material and is inversely proportional to its thickness. By increasing the material thickness, as well as the number of layers, the thermal insulation also increases due to the increased amount of trapped air. Therefore, it is necessary to determine the temperature gradients of individual layers; however, they were not determined in this study [7].

Pan studied heat loss as a function of environment temperature. He stated that the human body behaves as an internal heat source in a clothing system, thus establishing a temperature gradient between the indoor microclimate of the clothing and the environment. Depending on the environment temperature, which can be higher than, lower than or equal to the body temperature, the sign of the temperature gradient changes [8].

Xu studied the impact of multilayer protective clothing on thermal comfort. The multilayer protective clothing consisted of a T-shirt layer, a heat preservation layer, a PCM (phase change material) layer and an outer layer, with the PCM and heat preservation layers replacing places in combination. He analysed the temperature gradient between the subjects’ bodies, the multilayer protective clothing and the environment as a function of time. Based on the obtained temperature gradients, it was found that the PCM layer mainly absorbs the heat of the environment but also the heat of the human body when a person moves, which increases thermal comfort [9]. 

Yu et al. [10] investigated the impact of the thickness of an individual layer of clothing on the thermal properties but did not indicate the temperature gradients between the layers. However, they indicated the total temperature drop as a function of time. 

Temperature gradients have been used in examinations of the thermal insulation properties of naval constructions [11] and buildings [12]. Temperature gradients have also been used in other scientific fields and even in the study of the impact of temperature gradient metamorphism on the evolution of fabric in natural snow [13].

The review of the previous studies available in the literature shows that there is a need for new research on the thermal insulation properties of clothing, especially temperature gradients, and it is evident that the temperature gradients of composite clothing have not yet been measured. Therefore, the theoretical functional principles and the practical design of a new measurement method for the simultaneous determination of thermal resistance and temperature gradients in the design of thermal properties of clothing are presented in this paper. In the experimental part, practical measurements of thermal resistance and temperature gradients, obtained by measuring the mentioned parameters for built-in materials and textile material layers with the described measuring equipment, a thermal mannequin and sewn clothing items with the same textile material layers, are provided.

## 2. Theoretical Principles of the Method and Practical Design of the Measuring System

To describe the experimental implementation of the measurement procedure to determine the thermal resistance and temperature gradients in the design of thermal properties of clothing, the theoretical principles of the method are presented first, followed by the design features of the measurement system. A measuring system was set up under laboratory conditions with an analogue and then fully digital measuring method and computer data processing.

### 2.1. Stationary Heat Conduction as a Measurement Precondition

In many practical problems, such as in those related to textile materials and composite clothing, heat transfer through the solid takes place predominantly in one direction; e.g., in the x direction. Such cases occur especially when the dimensions of the solid (in the case of textiles and clothing, these are woven fabrics, knits, non-woven textiles, membranes and foils) are considerably larger in the other directions y and z (e.g., the length and width of flat textile products compared to their thickness) or when the heat flux in these directions is intentionally prevented by installing thermal insulation materials through which the heat conduction is very weak due to the low coefficient of thermal conductivity λ (e.g., the boundaries of working spaces in the measuring device). 

In the stationary state, the shape of the temperature field in the solid does not change; hence, each material point of the solid receives the same amount of heat from the warmer neighbouring material points and transfers the same amount to the colder neighbouring material points. The stationary state is described by the statement that Q = const. For such a state, the statement Q_x_ = Q const. applies approximately, while Q_y_ = 0 and Q_z_ = 0 are theoretical assumptions that are only possible with ideal insulation in the y and z directions.

### 2.2. Flat Textile Thermal Insulation Layer

**Figure 1:**
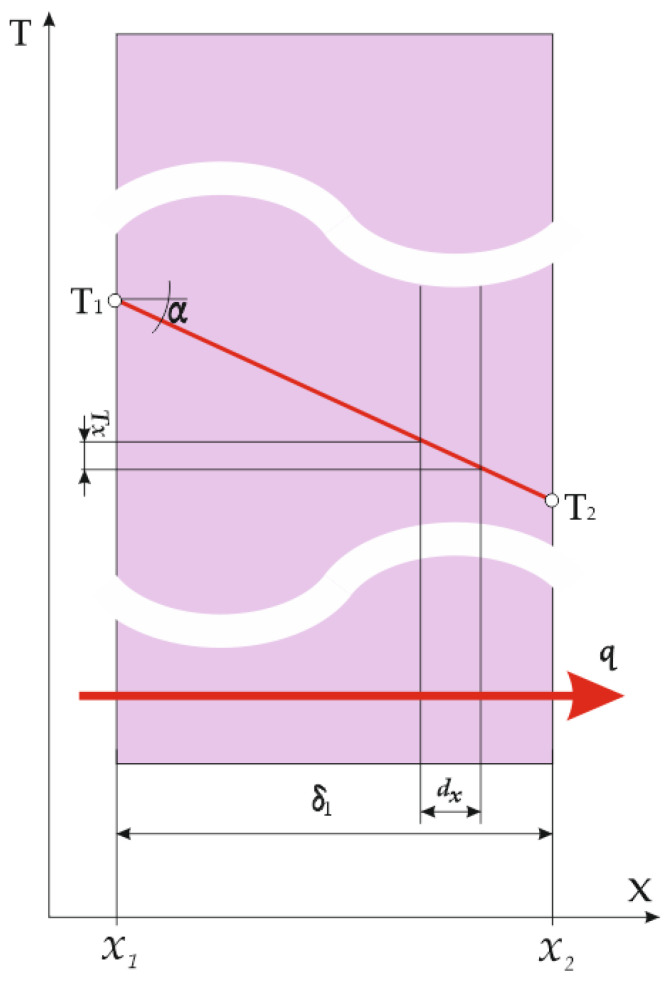
Temperature field for the flat thermal insulation layer for the case q = const.

It is characteristic of this physical model that the surface of the flat thermal insulation layer through which the heat flux passes is constant (A = const.) [14]. Since, in the stationary state, Q = const., in this case, q = Q/A = const. (Figure 1).

In accordance with the previously adopted assumptions that the entire surface of layer A1 at location $x_{1}$ has the same temperature T_1_ and the entire surface A_2_ at location $x_{2}$ has a temperature T_2_, the Fourier equation for a differential layer of thickness d_x_ is as follows [15]:

(1) $$q=\frac{Q}{A}=-\lambda \frac{dT}{dx}=const.$$

Considering that q = const. and assuming also that λ = const., Equation (1) can be integrated:

(2) $$q\overset{x_{2}}{\underset{x_{1}}{\int }}dx=-\lambda \overset{T_{2}}{\underset{T_{1}}{\int }}dT$$

(3) $$q\cdot \left(x_{2}-x_{1}\right)=-\lambda \cdot \left(T_{2}-T_{1}\right)$$

Since $x_{2}-x_{1}$= δ, the final result is:

(4) $$q=\frac{\lambda }{\delta }\cdot \left(T_{1}-T_{2}\right)$$

The order of the index is according to the mathematical rules, i.e., in the direction of the positive axis x, and therefore δ > 0, and the positive direction of the heat flux density vector q coincides with the x direction. This means that, in the case when *T_1_* < *T_2_*, the result would be q < 0, with the direction towards the negative axis x. Usually, in calculations, a higher temperature is entered at $T_{1}$ and a lower temperature at $T_{2}$ (both in °C or K), so that the result is q > 0 and the direction of the heat flux is clear from physical situation.

Equation (4) can be written in the form:

(5) $$q=\frac{\Delta T}{\frac{\delta }{\lambda }}$$

where ΔT *=* $\left(T_{1}-T_{2}\right)$ is the cause of heat transfer and (δ/λ) indicates the thermal resistance. 

The temperature gradient is a good indicator for assessing the insulating properties of a material. In accordance with the illustration in Figure 1, it can be represented as the tangent of the ratio of the temperature drop on the material surfaces ($T_{1}-T_{2}$) and the material thickness δ.

(6) $$\tan\alpha =\frac{\left(T_{1}-T_{2}\right)}{\delta }$$

In the case of textile materials, it can be concluded, according to Equation (6), that a material with a higher realized gradient tan α will be better as a thermal insulator; i.e., a higher temperature drop is achieved in the upper and lower material layers ($T_{1}-T_{2}$) with the smallest possible material thickness δ.

### 2.3. Flat Multilayer Composite Clothing

**Figure 2:**
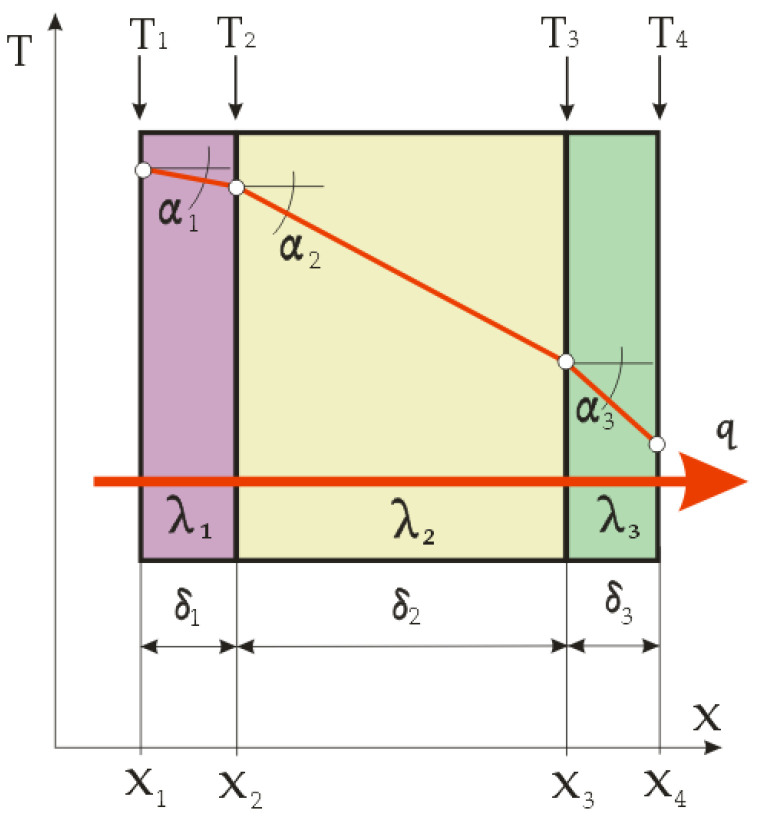
Temperature field in three-layer flat composite clothing (λ = const.).

The heat conduction through a multilayer flat composite can be determined by applying Equation (4) to each individual layer. For example, for the three-layer wall shown in Figure 2 (which is often used in clothing as a lining, thermal insulation and outer layer), three equations can be written [14]:

(7) $$q_{n}=\frac{\delta_{n}}{\lambda_{n}}\cdot \left(T_{n}-T_{n+1}\right)=q$$

By multiplying the equations by the corresponding (δ/λ) on the right-hand sides, only temperature differences remain. By summing up the equations, the temperatures between the layers *T_2_* and *T_3_* are cancelled and, after sorting, the following equation is obtained:

(8) $$q=\frac{T_{1}-T_{4}}{\frac{\delta_{1}}{\lambda_{1}}+\frac{\delta_{2}}{\lambda_{2}}+\frac{\delta_{3}}{\lambda_{3}}}$$

where the numerator indicates the difference between the temperatures of the outer surfaces and the denominator indicates the total thermal resistance.

For a multilayer flat composite with an arbitrary number of layers i = 2, 3, …, n and end temperatures $T_{1}$ and $T_{n+1}$, the following equation applies:

(9) $$q={\left(\overset{n}{\underset{i=1}{\sum }}\frac{\delta_{1}}{\lambda_{1}}\right)}^{-1}\cdot \left(T_{1}-T_{n+1}\right)$$

where the denominator on the right-hand side is the sum of the individual thermal resistances of all the composite layers.

Since each surface A_i_ has the same temperature T_i_ at all points, whatever it may be, the total heat flux can be calculated according to: Q = q_i_ · A_i_ = const.

The temperature gradients according to Figure 2 are:

(10) $$\tan\alpha_{n}=\frac{\left(T_{n}-T_{n+1}\right)}{\delta_{n}}$$

The calculation of the temperature gradients and the graphical representation of the temperature drop during the heat transfer through the layers, with their thicknesses plotted in proportion, very clearly show the thermal insulation significance of each textile material layer in the composite clothing. The best properties are exhibited by the thermal insulation layer with the gradient that has the highest values, i.e. which achieves the highest temperature drop during the heat transfer through it, and which at the same time has the lowest thickness.

### 2.4. Design of the Measuring System

**Figure 3:**
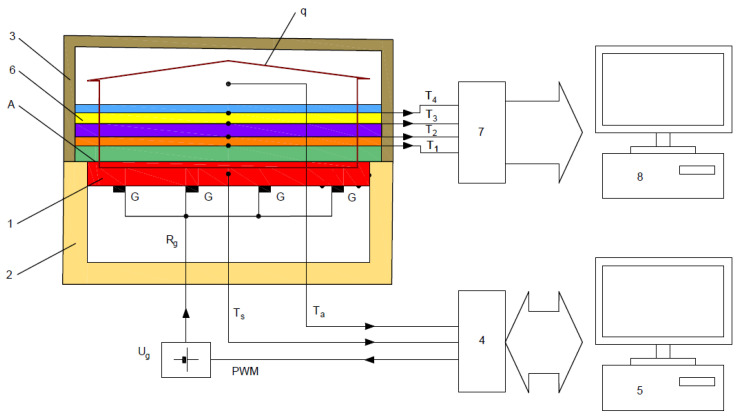
Schematic conceptual representation of the measuring system [16].

To determine the thermal characteristics of the textile material layers in composite clothing, a measuring system was used, the most important part of which is the measurement plate (position 1 in Figure 3), which is surrounded by the thermal insulation shield (2) and thermal insulation cover (3). 

The measurement plate with surface A must maintain a constant specified temperature and must be able to measure electrical power while maintaining the specified temperature. Therefore, the measurement plate is heated by several non-inductive point heaters G with total resistance R_g_, which are supplied by a voltage-stabilized source U_g_ of 48 V. The temperature of the measurement plate T_s_ is measured by a temperature sensor built into it and its value is outputted into a communication interface (4) with a built-in microcontroller. The achieved temperature is compared with the specified temperature in the computer (5). The computer regulates the electrical power required to maintain the specified temperature by dosing the power via the electrical control and determining the percentage of the control pulse-width (so-called pulse-width modulation (PWM)). Via the interface (4), the value of the ambient temperature Ta obtained from the temperature sensor located near the cover (3) is entered into the computer for calculation.

The measurement plate is made of 10-mm-thick aluminium. The plate is integrated into a metal block. The measuring surface (400 × 600 mm^2^) is surrounded by protective insulation, which prevents lateral heat flow from the edges of the sample. The relatively large area of the plate is not a problem with textile materials (they can be much larger surfaces), but the advantage is the reduction of the impact of heat losses at the edges of the layers.

First, the thermal resistance of the empty measurement plate must be determined. After a certain time, stable conditions are established, i.e., a stationary state of the heat flux q from the measurement plate towards the environment under the thermal insulation cover (3). The measurement plate temperature, ambient temperature and the electrical power supplied to the measurement plate, required to maintain the stationary state, are then read, and the thermal resistance of the empty measurement plate is determined with the expression:

(11) $$R_{ct0}=\frac{\left(T_{s}-T_{a}\right)\cdot A}{H_{0}}$$

where $R_{ct0}$ is the thermal resistance of the empty measurement plate (m^2^ K W^−1^); A is the measurement plate surface (m^2^); $T_{s}$ is the surface temperature of the measurement plate base (°C); T_a_ is the ambient temperature (°C); and H_0_ is the electrical power required to maintain the specified temperature of the measurement plate (W).

At the moment that thermal equilibrium is established (stationary state), $T_{s}$, $T_{a}$ and $H_{0}$ are constant and therefore q = const. Furthermore, the power required to maintain the thermal equilibrium of the measurement plate H_0_ can be calculated as follows:

(12) $$H_{0}=\frac{U_{g}^{2}\cdot P_{PWM}}{R_{g}}$$

where $U_{g}\;$ is the voltage of the stabilized source that supplies the non-inductive point heaters of the measurement plate; $P_{PWM}$ is the ratio of the PWM at the interface output (4); and $R_{g}$ is the total electrical resistance of the non-inductive point heaters.

The methodology of the measuring procedure was defined so that after measuring $R_{ct0}$, the textile material layers of the article of composite clothing (6) are organized in the order in which they will be placed in the garment, so that the first layer on the measurement plate is the one that will be closest to the body (most often the lining). This is followed by the laying of one or more thermal insulation layers, and the last outer layer is the outer shell. When laying the layers, the temperature sensors used to measure temperatures $T_{1}$ to $T_{4}$ are placed between each layer (Figure 3).

The general expression for calculating the thermal resistance in one or more layers of the composite clothing ($R_{ct}$) using the parameters shown, after restoring the stationary state ($T_{s}$ = const., $T_{a}$ = const., $H_{0}$ = const. and q = const.), takes the form of Equation (12):

(13) $$R_{ct}=\frac{\left(T_{s}-T_{a}\right)\cdot A\cdot R_{g}}{U_{g}^{2}\cdot P_{PWM}}-R_{ct0}$$

The measurement of the temperature gradients also begins after the establishment of the stationary state, and it proceeds in such a way that the temperature values $T_{1}$ to $T_{4}$ of the sensors placed between the textile material layers of the composite clothing are outputted to the measuring amplifier and temperature compensator at the cold end of the thermocouples (7), and then to the measuring computer (8). The steady state of the measured samples can be achieved in about 7 min. Due to the uniformity of the measurement condition, measurements of all measurement samples should start after 10 min. After that, measurement of the temperature gradients can take place every minute for 20 min, after which average values of the temperatures are calculated, similar to the thermal mannequins, according to ISO 15831. It is desirable for the measuring device without a cover (3) to be placed in the air-handling unit, together with the thermal mannequin, in order to ensure the same measurement conditions; alternatively, it can be constructed as a separate device with components determining the condition of the miniature air-handling unit under the cover (3) (Figure 3).

**Figure 4:**
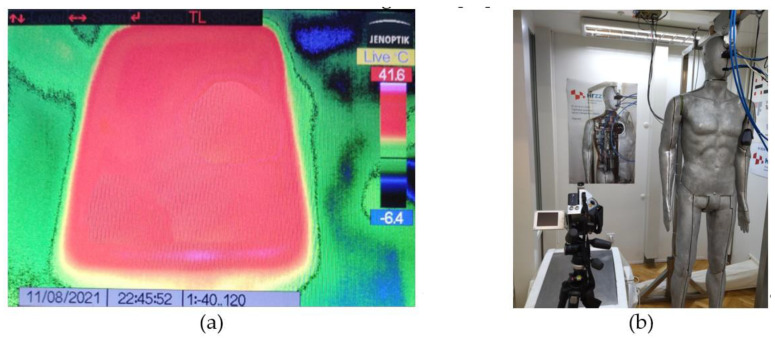
Examination of non-uniform temperature distribution in the casting surface using an IR camera: (**a**) the uniform temperature distribution in the casting surface measured using an IR camera; (**b**) the recording mode in the thermal chamber where the measurement plate and the thermal mannequin are located.

The use of non-inductive point heaters is important for this measurement method for several reasons: non-inductive point heaters allow for a variable installation geometry in the unit parts, thus achieving an even temperature distribution on the casting surface; i.e., a uniform infrared surface impression. A uniform temperature distribution can be ensured through an appropriate arrangement of the point heaters and by checking it with an infrared camera, such as a Jenoptik VarioCam [17] (Figure 4a). Checking for a uniform temperature distribution is carried out only for the first construction, where the measurement plate in the climate chamber, the measuring device and the thermal mannequin are located (Figure 4b) [18]. 

**Figure 5:**
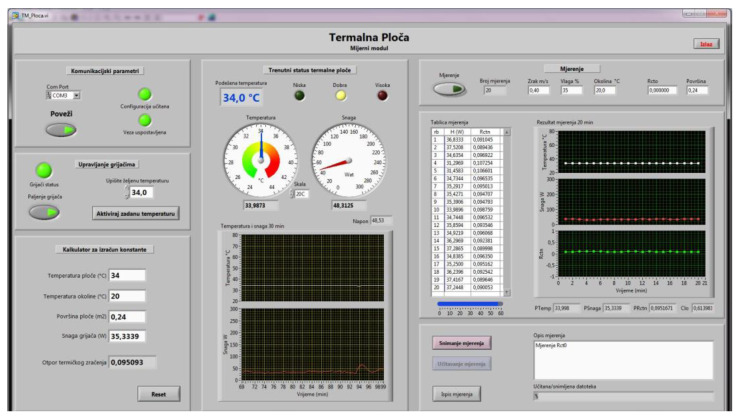
The display of the computer monitor for controlling and measuring the measurement plate.

Point heaters also allow complete conversion of the supplied electricity into heat, while in heaters formed from coiled wire in the form of solenoids, part of the supplied energy is converted into heat and part into an electromagnetic field due to the prominent inductive component at higher frequencies of PWM control. In addition, the pure ohmic resistance of non-induction point heaters does not distort the rectangular shape of the PWM pulse, which enables the accurate calculation of the electrical power dissipated through these heaters solely on the basis of the data known from the control, regulation and measurement system of the interface (4) (Figure 3). Thus, with this design, the commonly used instrumental methods for measuring electrical power are not required (use of a wattmeter, voltmeter and ammeter in the UI method of measuring power, etc.). Figure 5 shows the display of the computer monitor for controlling and measuring the measurement plate.

K-type thermocouples were chosen as temperature sensors because they have extremely small dimensions and practically do not affect the deformations or thickness changes in the textile material layers of composite clothing. The diameter of the measuring connection was only 0.3 mm, and the connection wires were 0.2 mm. These thermocouples are usable for temperatures ranging from −50 °C to 300 °C. The NI 9211 thermocouple temperature-measurement module with cold-end compensation together with the NI cDAQ-9172 from the National Instruments platform were selected for the measuring amplifier (Austin, TX, USA) [19,20].

## 3. Materials and Methods

**Figure 6:**
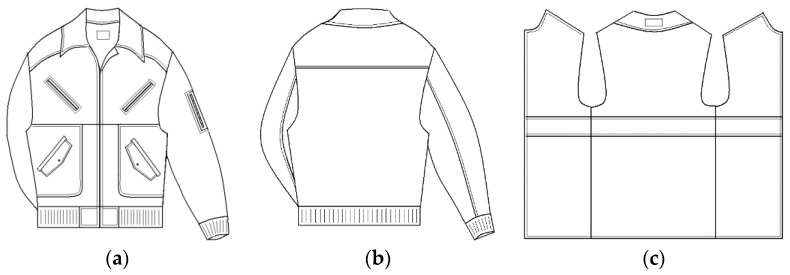
Sketch of the test jacket and thermal insert: (**a**) the front of the jacket; (**b**) the back of the jacket; (**c**) thermal insert.

For the purpose of performing an experiment that would prove the success of the new method and measuring device for simultaneous measurements of the thermal resistance in one or more textile material layers of composite clothing and the temperature gradients between the textile material layers, four layers of textile fabric products commonly used in the professional production of protective jackets were selected (Figure 6).

A fabric for the outer shell made of three-layer laminate designated as M3 (fabric upper-side: 100% polyester; membrane: polytetrafluoroethylene; fabric inner-side: 100% polyester fleece), a lining designated as M4 (100% polyester) and two types of thermal insulation material made of double-faced diamond-shaped quilted lining (DFDSQ lining) designated M5 (lining: 100% polyester; membrane: 100% polypropylene; padding: 100% polypropylene) and of fleece material designated M6 (100% polyester micro-fleece) were selected. The characteristic data for the fabrics used are given in Table 1, including material composition, mass per unit area, thickness δ and air permeability.

The test measurements included three groups: built-in materials, composite clothing and jackets.

In the first group, one layer of the built-in materials M1, M2, M3 and M4 of the protective jacket was tested in the horizontal position on the measuring plate, measuring thermal resistance and not the temperature gradients, but taking the temperatures of the measuring plate and the ambient air for the required values.

In the second group, different groups of textile material layers of composite clothing were formed in such a way that, for example, the lining was placed on the measuring plate first and then the other layers. Thus, the composite lining and the fabric of the outer shell were designated as OS11; the lining, the fleece thermal insulation insert and the fabric of the outer shell were designated as OS12; the lining, the thermal insulation insert made of DFDSQ lining and the fabric of the outer shell were designated as OS13; the fleece thermal insulation insert, the insulation insert of the DFDSQ-lining and the fabric of the outer shell were designated as OS14; and the thermal insulation insert made of the DFDSQ lining, the fleece thermal insulation insert and the fabric of the outer shell were designated as OS15. All groups of composite clothing articles were tested for thermal resistance in the horizontal position, and the temperatures between the textile material layers of the composite clothing were determined simultaneously in order to later determine the temperature gradients.

In the third group, the thermal characteristics of the textile material layers of composite clothing were examined after the practical integration of composites of the same composition, with the order of layers being OS11 to OS15, in five articles of clothing (protective jackets), with the measurement samples obtained from jackets designated as TM11 to TM15. A sketch of the model of the protective jacket is shown in Figure 6 along with a sketch of an additional thermal insulation insert which was zipped into the jacket. Textile material layers OS11 to OS15 were integrated into the jackets in a vertical position and tested on a thermal mannequin. The thermal resistance of each jacket was tested on the thermal mannequin according to ISO 15831.

A symbolic summary of the tests, indicating the measuring systems, positions, order, material type and the direction of the heat flux q, is provided in Table 2. The horizontal measurement plate was designated MP and the vertical part of the body of the thermal mannequin TM.

## 4. Results and Discussion

In accordance with the conditions presented in Section 3 of this paper, measurements of the thermal resistance on the measurement plate and the thermal mannequin and of the electrical power required to maintain thermodynamic equilibrium were recorded, the algebraic sum of all thermal resistances for all textile material layers in the composite clothing were calculated and the temperature gradients were determined, which are presented in Table 3. 

**Figure 7:**
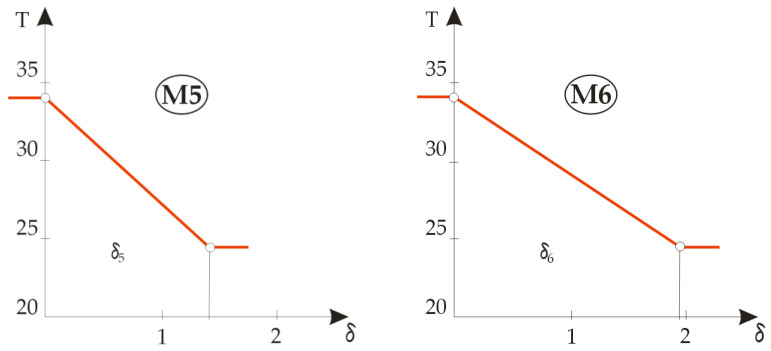
Graphical representations of the temperature gradients for built-in materials.

**Figure 8:**
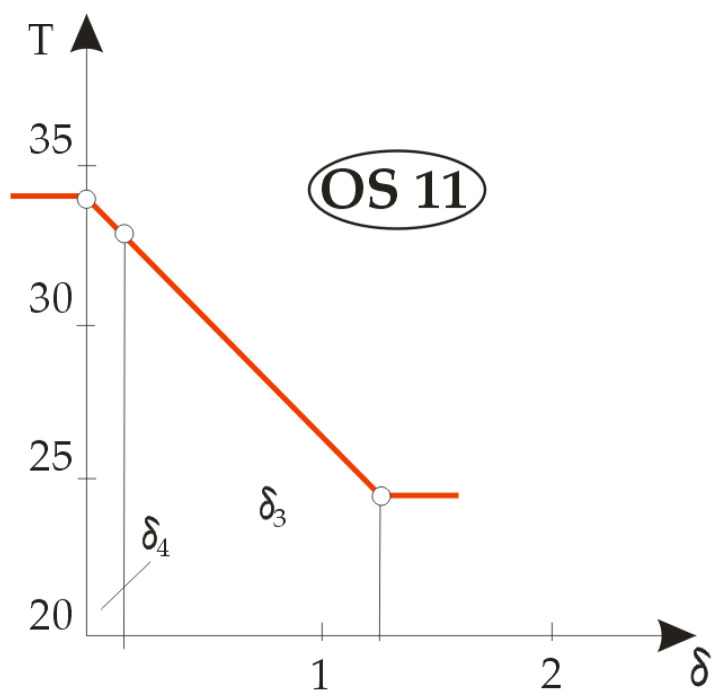
Graphical representation of the temperature gradient for an article of two-layer composite clothing.

**Figure 9:**
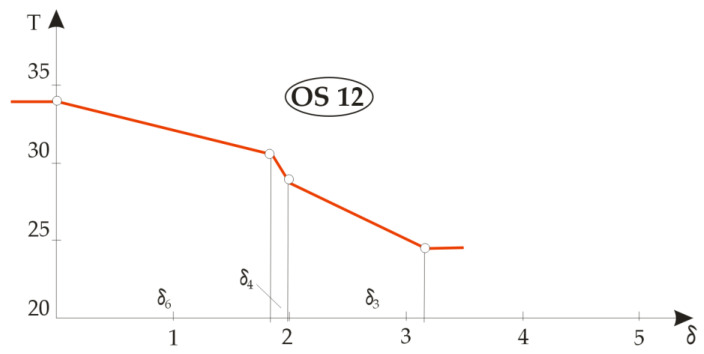

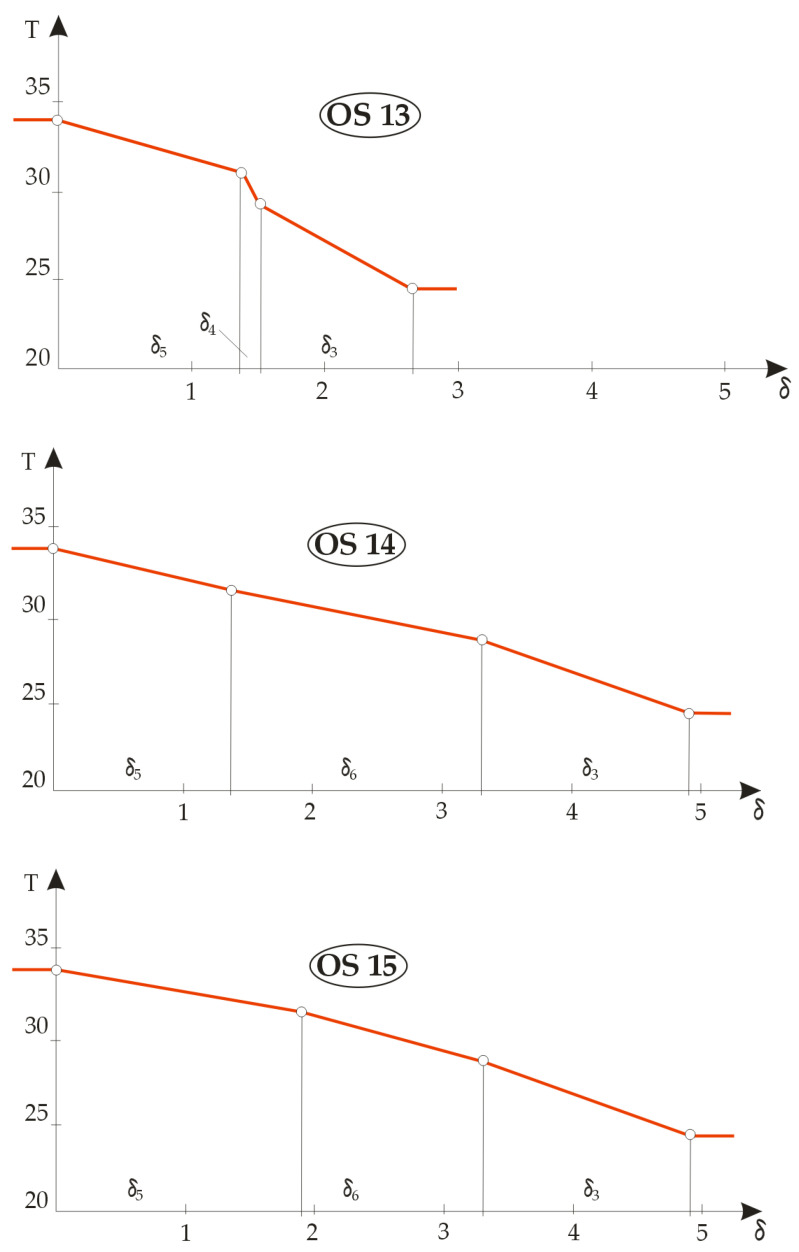
Graphical representations of the temperature gradients for different combinations of articles of three-layer composite clothing.

Based on the calculation results, three groups of temperature gradients were plotted: the first for the built-in components M1, M2, M3 and M4 ( Figure 7), the second for a two-element composite designated OS11 ( Figure 8) and the third for four different combinations of the three layers of textile material in Figure 9, designated OS12, OS13, OS14 and OS15.

In order to improve the process of the technical design of thermal clothing properties, a new measuring device was constructed and a new measuring method established that allow simultaneous measurements in the determination of the thermal resistance of one or more textile material layers of composite clothing, as well as for the temperature gradients between textile material layers. The device was designed in such a way that one part of the measuring device measures the thermal resistance with the help of a control measuring-computer and the other part of the device simultaneously measures the temperature gradients between the layers with the help of another measuring computer. Common to both parts is that the measurement is performed after thermodynamic equilibrium has been established; i.e., after a stable temperature in the measuring plate, a stable ambient temperature, a stable supply of electricity to the measurement plate (which is equal to the heat energy transferred to the measurement sample on the plate) and a stable heat flux from the plate through the test sample into the surrounding environment space have been established. The theory of how the device works is presented in Section 2.1, Section 2.2 and Section 2.3 of this paper, and the construction and features, as well as the measurement methodology, in Section 2.4.

As an example of the use of the new method, experiments were performed in which the thermal properties of a professional protective jacket were determined. For this, common materials often used in the manufacture of this type of jacket, namely the outer shell material, the lining and two types of thermal insulation material, were selected, the properties of which are shown in Table 1.

From these materials, five jackets, shown in Figure 6, were sewn with the same order of material integration as that in which the layers of the measurement samples of the composite clothing, intended for measurements on the measurement plate of the device, were formed. The thermal resistances and temperature gradients were measured in a horizontal position on the measurement plate and in a vertical position, built into the jacket, on a thermal mannequin, as shown in Table 2.

The measured results for the thermal resistance calculation for the built-in materials, the articles of composite clothing and the fabricated jackets, as well as the electric power required to maintain thermodynamic equilibrium, the temperature gradient calculation and the algebraic sum of the thermal resistance for all textile material layers in the composite clothing, are shown in Table 3.

Among the built-in materials, the lowest thermal resistance of 0.0005 m^2^ K W^−1^ was recorded for the lining designated M4, which was expected because its role was not thermal protection, and it was rather a thin material with a low coefficient of friction that made the jacket easier to wear and did not allow a view of the non-aesthetic interior of the jacket. The thermal insulation fabric upper-side M3 had a thermal resistance of 0.0099 m^2^ K W^−1^. The fleece material M6 had a high thermal resistance of 0.0324 m^2^ K W^−1^, and the DFDSQ lining M5 had the highest resistance of 0.0388 m^2^ K W^−1^. The power values required for maintaining thermodynamic equilibrium in the measurement samples gradually decreased from 20.43 W to 17.46 W.

Different combinations of the basic materials of the built-in components formed articles of composite clothing OS11 to OS15 (Table 2). It can be seen that the measured values of the thermal resistance gradually increased from 0.0202 to 0.1171 m^2^ K W^−1^, and the power at thermodynamic equilibrium in the measurement samples of the textile material layers designated dropped from 19.20 W for sample OS11 to 11.23 W for sample OS15.

Table 3 also shows the algebraic sums of the thermal resistance values. A comparison of the measured thermal resistance values in jackets OS11 to OS15 shows that the measured thermal resistance values were higher in jackets OS11 to OS15 than when the resistance values of the textile materials M3 to M6 in them were algebraically summed. This increase was highest for the jacket OS11 containing only the lining and the outer shell material at 94%, and it was lowest for the jacket OS14, composed of the DFDSQ lining M5, thermal insulation fleece material M6 and outer shell material M3, for which it was 30%. 

The observed increase in the thermal resistance value can be attributed to the formation of very thin air layers between the textile material layers due to the yarn crimp, which increased the overall thermal insulation properties of the composite clothing.

Measurements on the thermal mannequin were performed for sewn jackets, the internal structure of which was equal to the structure of the measurement samples OS11 to OS15. The lowest thermal resistance for a whole jacket (the weakest thermal insulation), 0.1832 m^2^ K W^−1^, was exhibited by the jacket designated TM11, which consisted only of the lining and the outer fabric, and 53.19 W was needed to maintain the thermodynamic equilibrium of the whole jacket. The highest thermal resistance (the best thermal insulation), 0.2986 m^2^ K W^−1^, was exhibited by the jacket designated TM14, which had a thermal insert made of the thermal insulation fleece material designated M6, DFDSQ lining M5 as the lining and the outer shell material M3. A power of 37.79 W was required to maintain the thermodynamic equilibrium of the jacket designated TM14. The thermal resistance values of the entire article of clothing when measured on a thermal mannequin were significantly higher than the measurement results on the measurement plate. There were several reasons for this. The first reason was that, when measured on a thermal mannequin, the textile material layers of articles of composite clothing were embedded in the clothing in a vertical position, so they could be separated, forming significantly larger air layers than when measured in a horizontal position. The second reason was the anatomical structure of the thermal mannequin itself, which was identical to the structure of the human body, so large air spaces were naturally created below the chest line on the chest and the back. The third reason was that the jackets themselves were constructed so that they had a large volume that allowed excellent ergonomics for movement and ease of wearing, which in turn had a favourable effect on the overall thermal insulation properties. Such large air spaces increased the thermal insulation properties of otherwise poorly thermally insulated clothing the most. Thus, with the jacket designated TM11, which had relatively modest thermal insulation properties, the total thermal insulation was found to increase by up to nine times, while in the well-designed jacket designated OS15 it increased by 2.5 times. It should be noted that the large relative increase in thermal insulation described should be critically considered through expert analysis of the absolute values of thermal resistance of individual articles of clothing. 

The presentation of a diagram with plotted temperature gradients can be a great help in designing the thermal properties of clothing because the most favourable thermal insulation materials can be identified visually. The most favourable thermal insulation material is the one with the largest gradient. Figure 7 shows the temperature gradients during the heat flux through the basic built-in materials designated M3 to M6. Since the temperature difference between the measurement plate and the ambient air is always the same for one material layer, this means that the gradient only depends on the material thickness, so the rule about the most favourable thermal insulation material cannot be adopted when measuring the gradient on a layer; the key data, however, are the measured values of the thermal resistance

Figure 8 shows the temperature gradients of the composite clothing designated OS11 from which the jacket designated TM11 was sewn, which consisted of only two layers: the lining and the basic outer fabric. It can be seen from the figure that, when analysing temperature gradients in two or more layers in an article of composite clothing, the rule is that the material with a higher temperature gradient has better thermal insulation properties. Due to the extremely small thickness, it is evident that the lining material had better thermal insulation properties, as indicated by a steeper gradient, but the outer fabric showed significantly better temperature-drop properties, such that almost the entire temperature drop was achieved in the outer fabric. Figure 9 shows the differences in temperature gradients for different combinations of three-layer composite clothing. The OS12 and OS13 systems were in fact OS11 double-layer jacket systems to which was added either a thermal insulation insert made of fleece material (M6), this system then being designated OS12, or a thermal insulation insert made of DFDSQ lining (M5), this system then being designated OS13. The temperature gradient diagrams look similar and in both the fractures of the curves are pronounced due to the thermal properties of the lining.

When the lining material M4 was replaced with a lining made of fleece material M6, with the thermal insert made of DFDSQ lining M5, the composite OS14 was obtained. A similar case occurred when the lining was replaced with M5 material and M6 was used as a thermal interlining, resulting in the composite fabric designated OS15. The temperature gradient diagrams in Figure 9 show no pronounced fractures and a uniform distribution of temperature drops across the textile material layers. It can be seen from the diagrams and measurement results that materials M5 and M6 had similar thermal insulation properties.

If the required thermal insulation can be obtained, and the analysis of the temperature gradients showed that some materials in the composite clothing had similar thermal insulation properties, a material with a lower cost, better visual appearance, lower surface area (and thus lower total weight) or pleasant feel or other preferred property should be selected.

From the results obtained, it can be seen that the algebraic sum of the temperature drops in the individual thermal insulation layers was equal to the difference between the temperatures of the measurement plate and the ambient air.

The reproducibility of the measurements was extremely high and the shedding was very low. The conditions for the measurement of the temperature gradients and the thermal resistance in the composite clothing were the same as the conditions for the measurement of the thermal insulation with the thermal mannequin (the measuring device and the thermal mannequin were in the same thermal chamber) in order to make the results as comparable as possible [18].

Based on the above, this paper presents the following novelties:

- Clothing technology does not have the long tradition of measurement and metrology techniques needed to test the properties of the materials required for the technical design of garments. Therefore, the introduction of a new measurement method for the simultaneous measurement of thermal resistance and temperature gradients is an important novelty for the field of clothing engineering. According to the previous studies available in the literature and a search of patent databases in the field of clothing engineering, there is no similar method, so the presented method can be considered a new one;
- One novelty is that the measurements of the two important parameters for the design of the thermal properties of clothing and textile material layers (thermal resistance and the values of the temperature gradients) can be carried out simultaneously with one measuring system;
- A further novelty is that the selected airflow velocity above the measuring sample was 0.4 m/s, while in other measuring systems it is 1 m/s. The reason for this is that, in the thermal mannequins with which the thermal insulation properties of the clothing were measured, an airflow velocity of 0.4 m/s and a temperature of 34 °C were determined according to the ISO standard, so that our method could ensure much better comparability of results for textile material layers and clothing than is possible, for example, with the method of measuring thermal resistance using the so-called hot plate;
- Our method and device can work separately or in combination with a thermal mannequin if the device is installed in the climate chamber with the mannequin, whereas this is not possible with the hot plate method;
- Another novelty is that our method, during the data acquisition, works according to a similar protocol as that with which the thermal mannequin works, so the results are more comparable from this point of view than with the hot plate method;
- A further novelty is that the existing hot-plate method can only measure thermal resistance, whereas our method measures two groups of parameters simultaneously;
- The next novelty is that the thermodynamic equilibrium established in our method can be detected when the stable value of the thermal resistance is reached, and this is when the measurement cycle of the temperature gradients begins;
- Another novelty is that our method uses K-type thermocouples, which have a measuring joint diameter of only 0.3 mm, so there is no deformation of the layers in the composite clothing (unlike with much thicker thermistors or resistance thermometers);
- An additional novelty is that we used a single circuit in which a multi-channel multiplex measurement amplifier, an A/D converter and a cold-end temperature compensator are integrated for each thermocouple;
- The unit can operate as an analogue unit (for simple measurements) and as a digital one with all functions;
- Our method does not require wattmeters to measure the power needed to maintain thermodynamic equilibrium but uses PWM and the equations given in the text to calculate power. This is a significant innovation that greatly simplifies the device design and reduces its costs;
- Another novelty is the use of non-inductive point heaters with variable geometry, allowing for a uniform temperature distribution on the measuring plate, and the application of PWM control technology;
- A further novelty is the speed and accuracy with which the thermal properties of complete composite clothing can be determined. Until now, this has been determined from experience;
- The final novelty concerns the determination of the influence of each layer on the temperature drop. If the layers have similar properties, this method makes it possible to choose a cheaper, more readily available or better material thanks to another property, which is not possible with other methods.

## 5. Conclusions

The presented measuring device for simultaneous measurements of the thermal resistance in one or more textile material layers of composite clothing and of the temperature gradients between the textile material layers of composite clothing, along with the new method for evaluating the determined parameters, can contribute to the engineering design of thermal properties of clothing. The clothing designer obtains a new device and method that allow them to have, to a greater extent than before, better insights into the thermal insulation properties of articles of composite clothing and into the selection of the most favourable layer from several available layers.

The research conducted on new measuring equipment with a measurement plate and measurements with a thermal mannequin has not only shown that the thermal insulation properties of textile material layers in articles of composite clothing can be algebraically summed, but that they are greater than the algebraic sum by between 30% and 94% due to the crimping and protruding fibres of the yarn, leading to the formation of thin air layers between the textile material layers, which increases the thermal insulation properties of the composite clothing. Furthermore, when the tested textile material layers were integrated in an item of clothing, they assumed a predominantly vertical position, with the distances between the layers being increased, and thus air layers of more pronounced widths appeared between the textile material layers of the composite clothing, and the thermal insulation properties of the integrated composites were increased (by between 2.5 and 9 times). The anatomical structure of the human body and the construction of clothing also contribute to this. It should be noted that these large increases are only relative ratios and that the real increase in thermal resistance depends, as a rule, on the increase in these parameters of the individual layers.

In contrast to the sum of the insulation properties of individual textile material layers of composite clothing, which is always less than the actual properties of the clothing, the algebraic sum of the temperature drops within the textile material layers is equal to the temperature difference between the temperature of the measurement plate and that of the ambient air.

For textile material layers of composite clothing, the layer with the best thermal insulation properties can be selected because it is characterized by the steepest temperature gradient; if two thermal insulation layers have similar thermal characteristics, the layer that is more favourable in terms of price or other parameters can be selected.

Likewise, the method is acceptable for clothing engineering, although it does not provide complete solutions. Therefore, as was noted, the sum of heat-resistance measurements is not the same when measuring textile material layers and when measuring the thermal properties of clothing made from these composite materials because air gaps occur. With this in mind, the presented method still has useful properties for clothing engineering with regard to the design of the thermal properties of clothing and the selection of the most favourable materials for thermal-insulation composite clothing. The mentioned measurements can be performed before the construction and production of clothing items, while on the thermal mannequin, measurements can be performed only when the clothing items have been sewn. This can be considered as another advantage of the presented method.

With the introduction of this new patented measuring device, clothing engineering gains a new measurement method for simultaneous measurement of thermal resistance and temperature gradients. 

The same parameters are measured in the same climate chamber according to the same protocol as for the thermal mannequin, so the results are comparable. 

Gradient measurements are initiated in our method after the established thermodynamic equilibrium is detected, when a stable value for the thermal resistance is reached. Measurements are carried out with very thin thermocouples, without deformation of the layers in the composite clothing, using PWM control and without the requirement for wattmeters. Using this method, experience has been replaced with scientific knowledge. The novelty is the ability to determine the influence of each layer on the temperature drop. If the layers have similar properties, this method makes it possible to choose a cheaper or more readily available material, or one with which is a better material thanks to another property, which is not possible with other methods.

## 6. Patent

Rogale, D., Firšt Rogale, S. and Knezić, Ž. Measurement equipment and method for the simultaneous determination of thermal resistance and temperature gradients of layers of composite clothing, No. P20211208A, The State Intellectual Property Office of the Republic of Croatia, 27. 7. 2021.

Rogale, D. and Nikolić, G. Measuring System for Determination of Static and Dynamic Thermal Properties of Composite and Clothing, No. PK20130350, The State Intellectual Property Office of the Republic of Croatia, 28. 8. 2015.

## Figures and Tables

**Table 1:** Characteristic Data for the Textile Fabrics used.

	M3	M4	M5	M6
Mass per unit area (kg m^−2^)	0.275	0.06	Lining: 0.11 × 2; membrane: 0.0125 × 2; padding: 0.0715 Total: 0.316	0.304
Thickness (mm)	1.16	0.09	1.40	1.90
Air permeability (mm s^−1^)	2.80	454.02	97.73	3.82

**Table 2:** Summary of the Tests Showing Measuring Systems, Test Positions, Order, Material Type and the Direction of the Heat Flux q.

Measurements on a Measuring Plate in a Horizontal Position		Measurements on a Thermal Mannequin (TM) in a Vertical Position	
1-ply construction materials	Multilayer composite construction	Protective jacket	
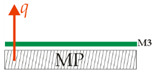 M3	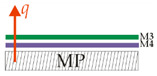 OS 11	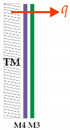	TM11
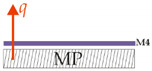 M4	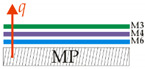 OS 12	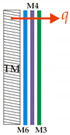	TM12
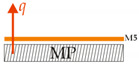 M5	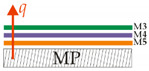 OS 13	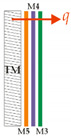	TM13
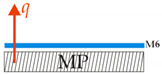 M6	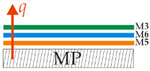 OS 14	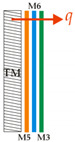	TM14
	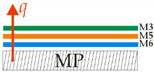 OS 15	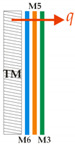	TM15

**Table 3:** Results for the Measurement and Calculation of the Thermal Resistance for Component Textile Materials, Layers of Composite Clothing and Realized Jackets; the Electrical Power Required to Maintain Thermodynamic Equilibrium, the Calculation of the Temperature Gradients and the Algebraic Sum of the Thermal Resistance for all Textile Material Layers in the Composite Clothing are also Shown.

Measurement system	Sample	Combination of Built-in Materials	R_ct_ (m^2^ KW^−1^)	P (W)	Gradient (°C/mm)	Algebraic Sum of Textile Material Layers, *R_ct_* (m^2^ KW^−1^)
Measurement plate	M3	-	0.0099	20.43	7.75	-
	M4	-	0.0005	22.01	100.6	-
	M5	-	0.0388	16.78	6.43	-
	M6	-	0.0324	17.46	4.74	-
	OS 11	M4 + M3	0.0202	19.20	11.1 6.8	0.0104
	OS 12	M6 + M4 + M3	0.0743	13.90	1.42 20.0 4.74	0.0428
	OS 13	M5 + M4 + M3	0.0850	13.18	1.66 21.1 4.97	0.0492
	OS 14	M5 + M6 + M3	0.1058	11.84	1.29 1.63 4.40	0.0811
	OS 15	M6 + M5 + M3	0.1171	11.23	1.32 2.64 3.28	0.0811
Thermal mannequin	TM11	M4 + M3	0.1832	53.19	-	-
	TM12	M6 + M4 + M3	0.2381	44.41	-	-
	TM13	M5 + M4 + M3	0.2425	44.93	-	-
	TM14	M5 + M6 + M3	0.2820	39.58	-	-
	TM15	M6 + M5 + M3	0.2986	37.79	-	-

## Data Availability

We choose to exclude this statement because the study did not report any data.
